# Morphological and genetic differences in legs of a polygamous beetle between sexes, *Glenea cantor* (Coleopter: Cerambycidae: Lamiinae)

**DOI:** 10.1371/journal.pone.0297365

**Published:** 2024-02-08

**Authors:** Jun Yan, Ping Luo, Yao Wu, Guandi Peng, Yini Liu, Chengrong Song, Wen Lu, Hongning Liu, Zishu Dong

**Affiliations:** 1 Jiangxi University of Chinese Medicine, Nanchang, China; 2 Jiangxi Provincial Department of Forestry, Nanchang, China; 3 Guangxi Key Laboratory of Agric-Environment and Agric-Products Safety, National Demonstration Center for Experimental Plant Science Education, College of Agriculture, Guangxi University, Nanning, Guangxi, China; Beni Suef University Faculty of Veterinary Medicine, EGYPT

## Abstract

The legs of insects play an important role in their daily behaviour, especially reproduction. Entomologists have performed much research on the role of the leg in different behaviours of beetles, an important group in the insect family, but relatively little has been done to study the ultrastructure and transcriptome of their legs. Hence, we systematically studied the ultrastructure and gene expression of the leg of *G*. *cantor*, a polygynous beetle, and compared its male and female diversity. In this study, we found the fore-leg, mid-leg and hind-leg of the female were significantly longer than those of the male. From the perspective of intuitive structural differences, we also compared the ultrastructures of the adhesion structure (tarsal) of males and females. The tarsal functional structure of the adult leg mainly includes sensilla and an adhesion structure. The sensilla on the tarsal joint mainly include sensilla chaetica (SCh II, SCh III) and sensilla trichodea (ST II). The adhesion structure includes disc-shaped bristles (di), lanceolate bristles (la), serrated bristles (se), spatula-shaped bristles (spl) and mushroom-shaped bristles (mus). Although there was no significant difference in sensillum distribution or type between males and females, there were significant differences in the distribution and species of adhesion structures between the fore-leg, mid-leg, and hind-leg of the same sex and between males and females. Therefore, different adhesion structures play different roles in various behaviours of beetles. On the other hand, the transcriptome results of male and female legs were screened for a subset of olfaction- and mechanics-related genes. We discovered that the male leg showed upregulation of 1 odorant binding protein (OBP), 2 Olfactory receptors (ORs) and 2 Chemosensory proteins (CSPs). Meanwhile, the female leg showed upregulation of 3 OBPs, 1 OR, 1 Gustatory receptor (GR) and 3 Mechanosensitive proteins (MSPs). An in-depth examination of the ultrastructure and molecular composition of the legs can elucidate its function in the reproductive behavior of *G*. *cantor*. Moremore, this investigation will serve as a cornerstone for subsequent research into the underlying behavioral mechanisms.

## Introduction

Crawling and walking are basic forms of locomotion in animals, and insects, the largest group of animals, have naturally mastered these skills. Over a long period of evolution, insects have developed a high level of crawling and attaching ability, being able to walk not only on horizontal surfaces but also on vertical surfaces, which is the basis of their everyday behaviour. This ability of insects to crawl and attach is largely dependent on the high degree of leg development, especially the fine functional adhesion system [1]. Humans have been interested in the ability of insects to crawl and adhere to smooth surfaces for centuries. Initially, the general morphology of the fly’s leg was observed and described by museum scientists using a primitive light microscope. With the invention and widespread use of electron microscopy, it has become possible to make precise observations of the external morphology and internal structure of research objects at the micro- and nanoscales [2]. At present, studies on the attachment mechanisms of insect legs are mainly performed on Orthoptera [3], Hemiptera [4], Hymenoptera [5], Diptera [6] and Phasmatodea [7].

In addition, with the gradual development of experimental instruments and science and technology, research on insects has gradually transitioned from a focus on basic structure, external morphology, receptor classification, etc., to a focus on physiological and biochemical processes, genetic and receptor internal composition and related proteins [8–11]. With the rapid development of next-generation sequencing technology for transcriptome analysis, a considerable number of scholars have identified and classified genes and related proteins in insects and connected them with their daily life and reproductive behaviours [12–14]. Insects have chemoreceptors; for instance, numerous studies have found that they are located within specialized sensilla, most of which are scattered on the antennae, mouthparts, legs, and oviposition organs [15–19]. These chemoreceptors play a crucial role in the contact chemoreception of nonvolatile chemicals. However, similar studies on the legs of the beetle family are scarce in this area.

Cerambycidae, one of the main groups of the order Coleoptera, includes economically important pests of street trees and forests, with more than 36,000 described species worldwide [20]. Glenea cantor Fabricius is a longhorn beetle whose larvae bore under the bark of living trees of at least seven plant families in Southeast Asia, entering the wood for pupation [21,22]. Their reproductive behaviour has been systematically studied [23,24], but we do not yet know how the differences in the structure and genetics of legs between sexes relate to behavioural differences.

Thus, the purpose of this study was to first observe the differences between sexes in the morphology and adhesion structures of the legs of adult *G*. *cantor* via SEM and then analyse the genetic differences between male and female legs by transcriptomics. By assessing the differences between sexes in the adhesion structures and genetics of the legs, some behaviours, such as selective mating and ovipositing behaviour, will be further understood. The physiological functions of these sensilla may provide an effective control target for this pest. These results should lay a foundation for an in-depth understanding of the olfactory mechanism of *G*. *cantor* and provide a reference for further research on the prevention and control of longhorn beetles through olfactory mechanisms.

## Materials and methods

### Insects

In June 2020, we collected three kapok plants from Qingxiu Mountain (22°12’-23°32’ N, 107°45’-108°51’ E), Nanning, China, that had withered in the upper stalk due to *G*. *cantor* infestation and cut them into 10 sections (10–15 cm in diameter × 40 cm in length). The stalk sections were placed in four bubble containers in a yarn cages (40 cm × 80 cm × 80 cm) sprayed with water once every 7 d in a controlled environment (25 ± 1°C, 70 ± 5% RH, 14 L: 10 D) [24]. After 1.5 mo, newly emerged adults from the cages were randomly collected and transferred to separate glass containers (4 cm in diameter × 12 cm in length). These adults were fed with kapok twigs (2–4 cm in diameter × 8 cm in length), which were renewed every 2 d.

### Preparation of Specimens for SEM

We selected five male and five female adults of *G*. *cantor* and placed them in a freezer at −20°C. After 30 min, the adults were removed and cut separately according to the fore-leg, mid-leg and hind-leg under a stereomicroscope PX-1 (Camsonar Technology Co., Ltd., Beijing, China). The legs were stored in a 70% alcohol solution until they were examined.The legs were cleaned three times in an ultrasonic bath JP-010T (Skymen Cleaning Equipment CO., Ltd., Shenzhen, China) at 250 W for 300 s each. The legs were then fixed separately in 2.5% glutaraldehyde at 4°C for 12 h. The legs were dehydrated through an ascending ethanol series of 75%, 80%, 85%, 90%, 95%, and 100% with 10 min intervals. The prepared legs were stored in a glass container with a desiccant and dried for 24 h. After drying, the samples were mounted on a holder using double-sided sticky tape (dorsal, ventral), sputter coated with gold-palladium, and then observed under a SEM (model S-3400 N, Hitachi, Japan) operated at 5–10 kV. Images were digitally recorded and stored on a computer.

### Preparation of Specimens for transcriptome

Tissue expression was obtained from legs of 30 d *G*. *cantor* adults of different sexes (a group of 10 adults) were prepared for RNA extraction. Three biological replicates were performed per group. Samples were excised on ice and immediately frozen in liquid nitrogen, and stored at −80°C until RNA isolation.

A NanoPhotometer spectrophotometer (Thermo Fisher Scientific, Massachusetts, USA) and the Nano6000 Assay Kit for the Agilent Bioanalyzer 2000 system (Agilent Technologies, California, USA) were applied to check the purity and integrity of the total RNA, respectively. After total RNA extraction, magnetic beads with Oligo dT (Thermo Fisher Scientific, Hampton, USA) were used to enrich mRNA. Then, cDNA fragments were purified using the QiaQuick PCR extraction kit (Qiagen, Venlo, The Nether-lands), ends repaired, A bases added and ligated to Illumina sequencing adapters. The ligation products were screened by agarose gel electrophoresis and amplified by PCR and RNA sequencing was performed using novaseq 6000 (Illumina) by Novogene Biotech Co. (Beijing, China).

### Morphology data analysis

The identification and classification of the sensillum types and the terminology used in this work were based on the studies of Schneider [25], the definition of adhesive structure was referred to Beute and Gorb [26]. The sensillar and adhesive structure distribution patterns were precisely described using Adobe Photoshop software, Version CS6. The distribution of each type of sensillum and adhesive structure was analyzed between the Three pairs of legs in both sexes.

In all, five individual structures per leg type were subjected to a length analysis of legs. The t-test was applied to determine the possible sexual dimorphism of tarsus between males and females using the SPSS statistical software package, version 25.0 (SPSS Inc., Chicago, IL). Student’s t-test or analysis of variance were applied to determine the possible differences in of the length of tarsus, and the values are reported as means ± standard error (SE). The significance level was set at 0.05.

### Transcriptome data analysis

The sequencing library was constructed using high-quality RNA and sequenced using DNBSEQ-T 7 (2 × 150 bp read length). The transcriptome data analysis method was referred to Wu (2023). The *G*. *cantor* CSP, GR, OR, OBP and MSP nucleotide sequences were used as queries (BLASTx) in the GenBank database (http://www.ncbi.nlm.nih.gov/BLAST/) with an E-value threshold of 1e-5 for the NCBI nonredundant protein (Nr) database (http://www.ncbi.nlm.nih.gov), the Swiss-Prot protein database (http://www.expasy.ch/ sprot), the Kyoto Encyclope-dia of Genes and Genomes (KEGG) database (http://www.genome.jp/kegg). Protein functional annotations could then be obtained according to the best alignment results.

## Results

### Gross morphology of adhesion organs

**Fig 1:**
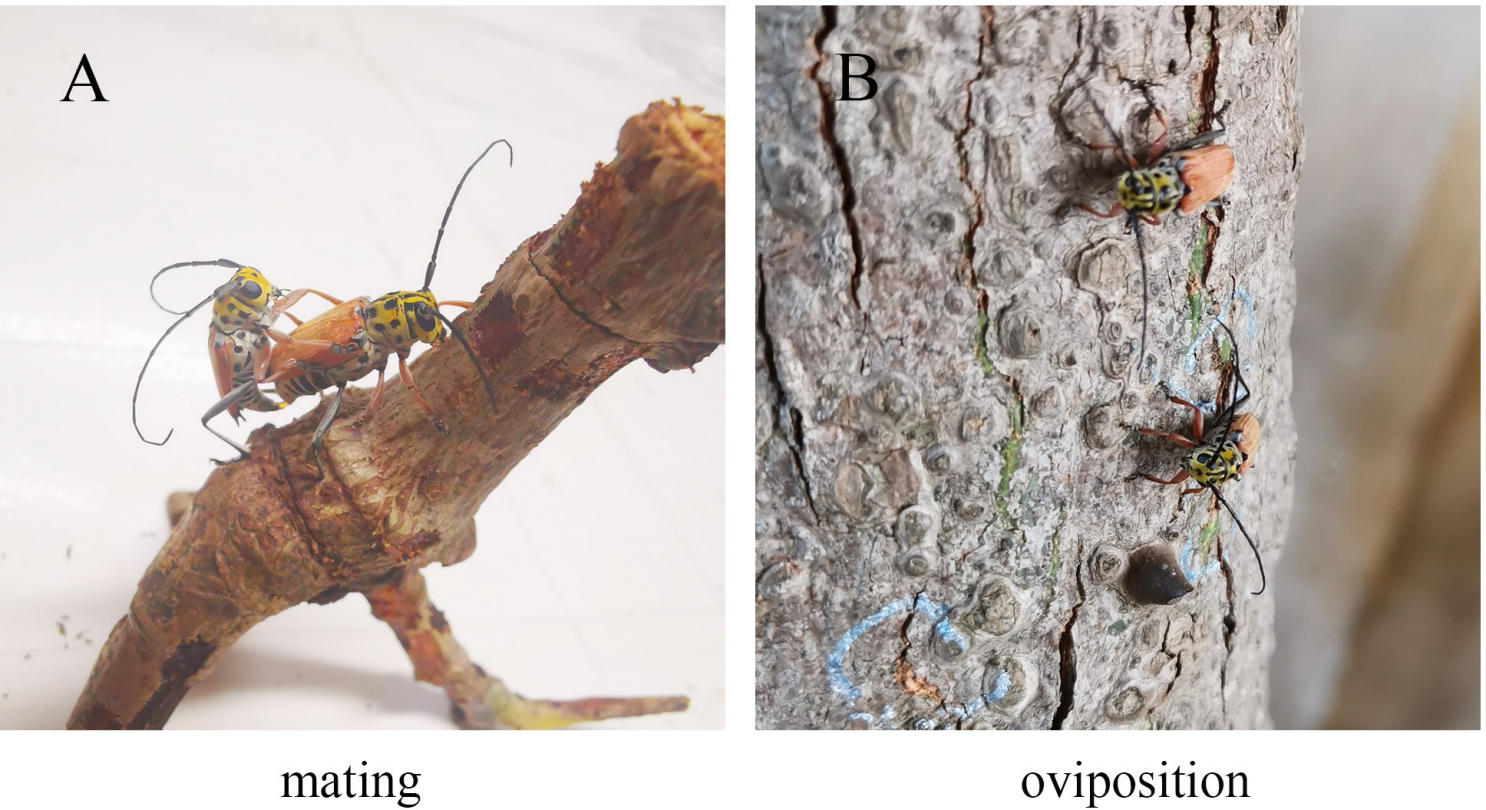
Photograph of oviposition and mating of *G*. *cantor*.

All the legs of *G*. *cantor* are typically walking legs. There was clear male/female dimorphism in the length of the four developed parts of the trochanteral, femoral, tibial and tarsal segments, except in the coxa (Table 1). The tarsal segments are all relatively well developed, with the first three femoral tarsal segments specializing as bristle-type claw pads and the fore tarsal segments forming two lateral claws. This dimorphism in tarsal segments is directly related to the functional differences in the daily behaviour of male and females (Fig 1).

**Table 1:** The lengths of the different sections of legs of both sexes in *G*. *cantor*.

Part	Designation	Length (mm)		Statistical data		
		♀	♂	t	P	
Fore-leg	Coxa	0.38±0.01	0.38±0.04	0.09	0.931	ns
	Trochanter	0.44±0.00	0.41±0.01	3.44	0.040	*
	Femur	3.30±0.03	2.70±0.03	15.06	0.000	***
	Tibia	3.04±0.04	2.59±0.02	10.05	0.000	***
	Tarsus	1.72±0.03	1.49±0.03	5.34	0.000	***
Mid-leg	Coxa	0.38±0.00	0.38±0.01	0.73	0.482	ns
	Trochanter	0.39±0.00	0.41±0.00	-2.16	0.045	*
	Femur	3.47±0.02	3.20±0.09	2.93	0.016	*
	Tibia	3.28±0.03	2.72±0.03	13.04	0.000	***
	Tarsus	1.85±0.04	1.68±0.06	2.46	0.024	*
Hind-leg	Coxa	0.37±0.03	0.36±0.02	0.40	0.694	ns
	Trochanter	0.42±0.02	0.32±0.00	4.67	0.001	**
	Femur	4.80±0.03	4.32±0.05	7.68	0.000	***
	Tibia	4.53±0.04	4.20±0.08	3.76	0.002	**
	Tarsus	1.97±0.05	1.82±0.04	2.22	0.040	*

Note: Length measurements are centreline measurements. Data are presented as the means ± SEs, n = 10.

### Ultrastructural morphology of legs

**Fig 2:**
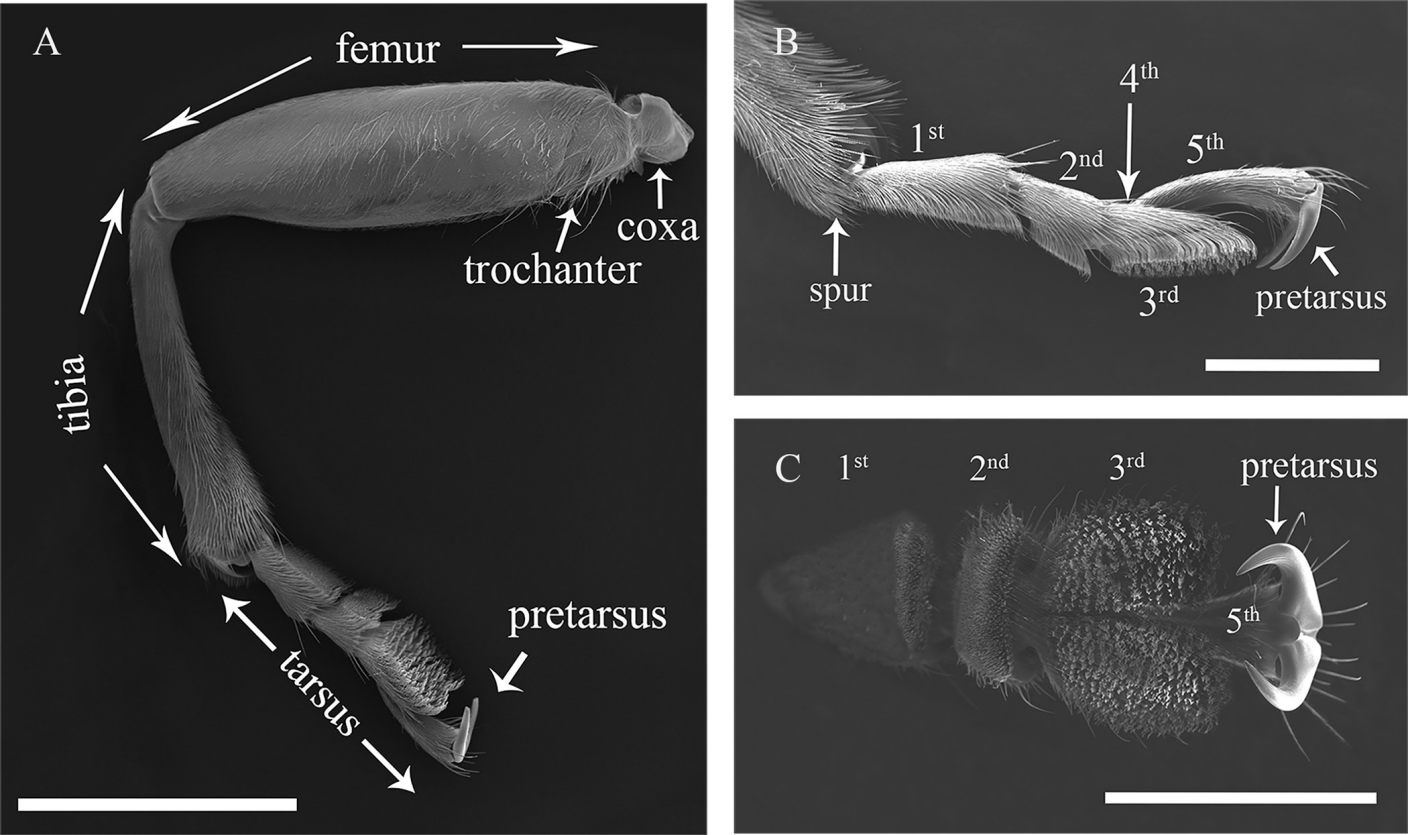
The ultrastructure of the legs of *G*. *cantor*. A, Lateral view of the fore-leg of a female; B, Lateral view of the hind-leg of a female; C, Ventral view of the hind-leg of a female. Scale bars: A = 2 mm; B, C = 1 mm.

The landing part of legs of the longhorn beetle includes the anterior tarsal joint, the tarsal joint and the end of the tibial joint. The anterior tarsal joint is double hooked, and the tarsal joint is mainly divided into 5 segments. The end is connected to the anterior tarsal joint, and the base is connected to the tibial joint. These structures are located at the end of the tibial joint (Fig 2).

**Fig 3:**
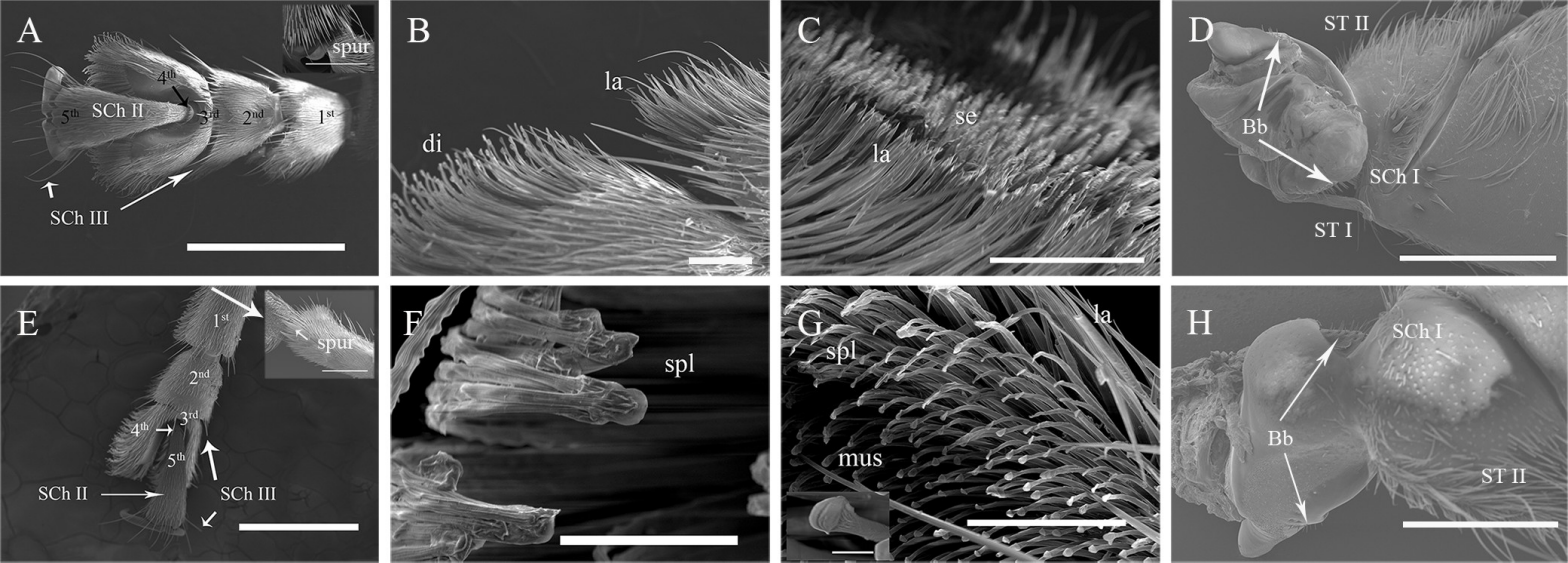
Ultrastructure of the tarsus of the fore-leg of adult *G*. *cantor*. A, Dorsal view of the tarsus of the fore-leg of a female; B, The junction of the 2nd-3rd tarsi of the fore-leg of a female; C, The 2nd tarsus of the fore-leg of a female; D, The femur of the fore-leg of a female; E, Dorsal view of the tarsus of the fore-leg of a male; F, A lanceolate bristle on the 3rd tarsus of the fore-leg of a male; G, A mushroom-shaped bristle on the 1st tarsus of the fore-leg of a male; H, The femur of the fore-leg of a male. Scale bars: A, E = 1000 μm; D, e = 500 μm; a = 400 μm; B, C, G = 100 μm; F = 30 μm; g = 5 μm.

**Fig 4:**
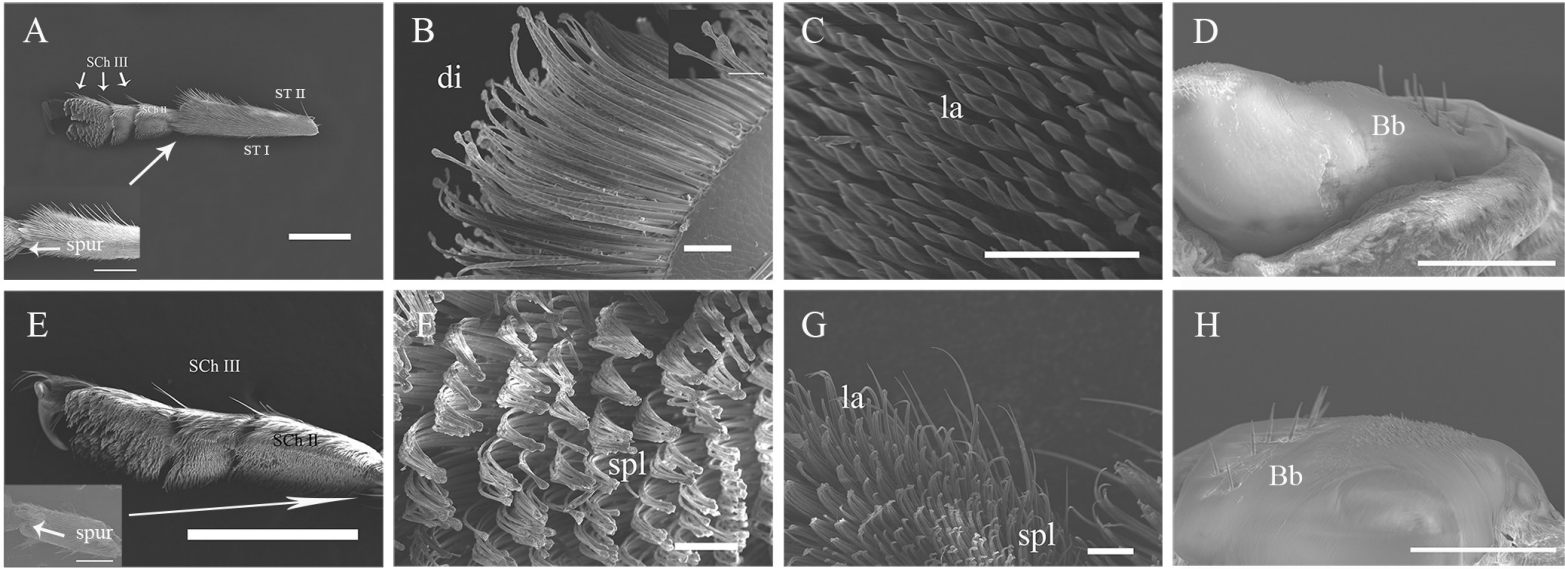
Ultrastructure of the tarsus of the mid-leg of adult *G*. *cantor*. A, Venter view of the tarsus of the mid-leg of a female; B, A discoid bristle on the 3rd tarsus of the mid-leg of a female; C, A lanceolate bristle on the 1st tarsus of the mid-leg of a female; D, The femur of the mid-leg of a female; E, Venter view of the tarsus of the mid-leg of a male; F, A spatula-like bristle on the 3rd tarsus of the mid-leg of a male; G, Venter view of the 2nd tarsus of the mid-leg of a male; H, The femur of the mid-leg of a male. Scale bars: A, E = 1000 μm; a, e = 500 μm; D, H = 100 μm; B, C, F, G = 50 μm; b = 30 μm.

**Fig 5:**
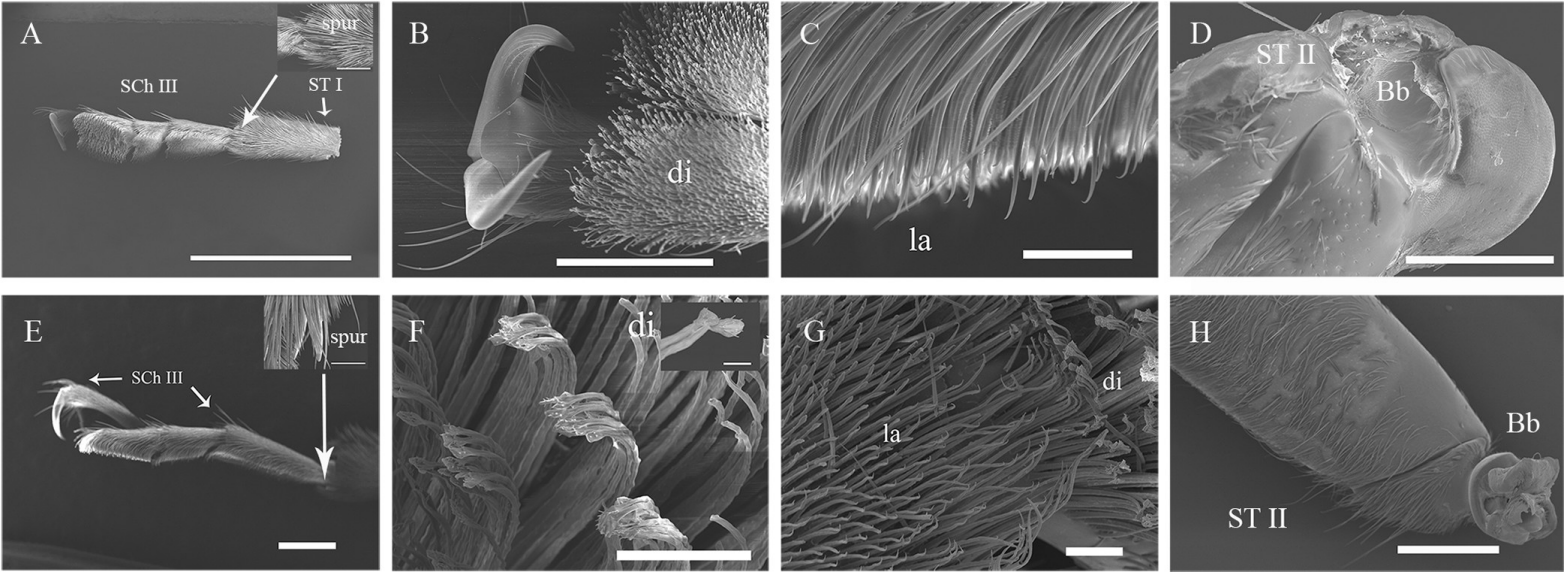
Ultrastructure of the tarsus of the hind-leg of adult *G*. *cantor*. A, Lateral view of the tarsus of the hind-leg of a female; B, Venter view of the 3rd tarsus of the hind-leg of a female; C, Lateral view of the 1st tarsus of the hind-leg of a female; D, The femur of the hind-leg of a female; E, Lateral view of the tarsus of the hind-leg of a male; F, A discoid bristle on the 3rd tarsus of the hind-leg of a male; G, The junction of the 2nd-3rd tarsi of the hind-leg of a male; H, The femur of the hind-leg of a male. Scale bars: A, E, H = 500 μm; D, a = 300 μm; e = 100 μm; B, C, F, G = 50 μm; f = 10 μm.

Similar to the order and mechanism of walking from the heel to the tiptoe of the legs in humans, the spur of *G*. *cantor* lands first, which is distributed on the tibia (Tables 2–4; Figs 2B, 3A, 3E, 4A, 4E, 5A and 5E). Thereafter, the tarsal segments land in the sequence 1st→2nd→3rd. Then, the contact launches adhesion, and finally, the two hooks that connect the 5th tarsus are directly hooked into the surface to finally fix the landing point. Similarly, when *G*. *cantor* needs to disconnect from the surface, the contact process is performed in reverse order. The 4th, 5th and front segments form an arched bridge (Fig 2A), so the 4th and 5th sections do not directly contact the landing surface.

**Table 2:** Different structures and sensillum distributions on the tarsus of the fore-leg of adult *G*. *cantor*.

Type	Designation	Subtype	Sex	Coxa		Trochanter		Femur		Tibia		Tarsus													
												1^st^			2^nd^			3^rd^			4^th^			5^th^	
				V	D	V	D	V	D	V	D	V	D		V	D		V	D		V	D		V	D
Sensilla	Böhm bristles	Bb	♀	+	+																				
			♂	+	+																				
	Sensilla Chaetica	SCh I	♀			+		+		+	+														
			♂			+		+		+	+														
		SCh II	♀					+	+	+	+		+			+			+			+		+	+
			♂					+	+	+	+		+			+			+			+		+	+
		SCh III	♀								+		+			+			+			+		+	+
			♂								+		+			+			+			+		+	+
	Sensilla Trichodea	ST I	♀							+	+		+			+			+			+			+
			♂							+	+		+			+			+			+			+
		ST II	♀				+		+																
			♂				+		+																
Spur	Spur	Spur	♀							+															
			♂							+															
Adhesion	Discoid bristle	di	♀															+							
			♂																						
	Lanceolate	la	♀									+			+										
	bristl		♂									+			+										
	Serrated	se	♀												+										
	bristle		♂																						
	spatula-like	spl	♀																						
	bristle		♂												+			+							
	Mushroom-	mus	♀																						
	shaped bristle		♂									+													

Note: “+” indicates the presence of the functional structure, and a blank indicates that the functional structure is absent. Same as the following Tables 3 and 4.

**Table 3:** Different structures and sensillum distributions of the tarsus of the mid-leg of adult *G*. *cantor*.

Type	Designation	Subtype	Sex	Coxa		Trochanter		Femur		Tibia		Tarsus													
												1^st^			2^nd^			3^rd^			4^th^			5^th^	
				V	D	V	D	V	D	V	D	V	D		V	D		V	D		V	D		V	D
Sensilla	Böhm bristles	Bb	♀	+	+																				
			♂	+	+																				
	Sensilla Chaetica	SCh I	♀			+		+		+	+														
			♂			+		+		+	+														
		SCh II	♀					+	+	+	+		+			+			+			+		+	+
			♂					+	+	+	+		+			+			+			+		+	+
		SCh III	♀								+		+			+			+			+		+	+
			♂								+		+			+			+			+		+	+
	Sensilla Trichodea	ST I	♀							+	+		+			+			+			+			+
			♂							+	+		+			+			+			+			+
		ST II	♀			+		+																	
			♂			+		+																	
Spur	Spur	Spur	♀							+															
			♂							+															
Adhesion	Discoid bristle	di	♀															+							
			♂																						
	Lanceolate	la	♀									+			+										
	bristl		♂									+			+										
	Serrated	se	♀												+										
	bristle		♂																						
	spatula-like	spl	♀																						
	bristle		♂												+			+							

**Table 4:** Different structures and sensillum distributions of the tarsus of the hind-leg of adult *G*. *cantor*.

Type	Designation	Subtype	Sex	Coxa		Trochanter		Femur		Tibia		Tarsus													
												1^st^			2^nd^			3^rd^			4^th^			5^th^	
				V	D	V	D	V	D	V	D	V	D		V	D		V	D		V	D		V	D
Sensilla	Böhm bristles	Bb	♀	+	+																				
			♂	+	+																				
	Sensilla Chaetica	SCh I	♀			+		+		+	+														
			♂			+		+		+	+														
		SCh II	♀					+	+	+	+		+			+			+			+		+	+
			♂					+	+	+	+		+			+			+			+		+	+
		SCh III	♀										+			+			+			+		+	+
			♂										+			+			+			+		+	+
	Sensilla Trichodea	ST I	♀							+	+		+			+			+			+			+
			♂							+	+		+			+			+			+			+
		ST II	♀			+		+																	
			♂			+		+																	
Spur	Spur	Spur	♀							+															
			♂							+															
Adhesion	Discoid bristle	di	♀															+							
			♂															+							
	Lanceolate bristl	la	♀									+			+										
			♂									+			+										

The functional structure of the legs of *G*. *cantor* mainly includes 3 categories: spur, sensilla and adhesion structure (Tables 2–4; Figs 3–5). The sensillum types include Böhm bristles (Bb), sensilla chaetica (SCh I, SCh II, SCh III) and sensilla trichodea (ST I, ST II). Meanwhile, the adhesion structure includes disc-shaped bristles (di), lanceolate bristles (la), serrated bristles (se), spatula-like bristles (spl) and mushroom-shaped bristles (mus).

### Böhm bristles

Böhm bristles (Bb) are a special type of spinous sensillum that are distributed at the junction of the body and legs. They are distinctly distributed ventrally and dorsally at the base of the coxa of the fore-legs, mid-legs and hind-legs of both males and females (Tables 2–4; Figs 3–5).

### Sensilla chaetica

Sensilla chaetica are widely distributed on the legs. These sensilla are more robust than other types of sensilla. These sensilla could be further classified into three subtypes based on length and shape: SCh I, II, and III. There were no statistically significant differences observed in the distribution on the legs, either between males and females or among the fore-legs, mid-legs, and hind-legs.

- Sensilla chaetica type I (SCh I) are cone-shaped and attached to the surface of the legs (Figs 3–5). They are mainly spread all over the tibiae of different legs of males and females. Meanwhile, they are also found on the venter of the trochanter and femur (Tables 2–4).
- Sensilla chaetica type II (SCh II) are clearly recognizable, and their tips protrude beyond all other sensilla. These sensilla are at nearly a 45° angle to the surface of the legs (Figs 3–5) and are long, robust, and slightly curved hairs with a blunt tip. SCh II are widely distributed on the legs, except the coxa and trochanter, and their distribution on the tarsus is restricted to the dorsal side (Tables 2–4).
- Sensilla chaetica type III (SCh III) are abnormally developed. They are stout and straight and distributed at the junction of several sections of the legs, especially the tarsus (1st - 5th) and tibia (Tables 2–4).

### Sensilla trichodea

Sensilla trichodea are hair-like protruding receptors and are widely distributed on the antennae. The base of each sensillum is embedded in a wide or narrow receptor nest, and the morphological structure of sensilla trichodea can be classified into two distinct types: ST I and II. There was no significant difference in the distribution between sexes or for each leg.

- Sensilla trichodea type I (ST I)are among the most commonly reported sensilla. The setae on the legs of ST I exhibit a curved shape with blunt tips, and they are tightly inserted into small cuticular sockets at an angle of 45–60° relative to the legs surface (Figs 3–5) (Tables 2–4).
- Sensilla trichodea type II (ST II) are inserted into broad sockets; they are long, slender, and straight or curved with smooth surfaces (Figs 3–5). ST II are present only on the trochanter and femur of the legs (Tables 2–4).

### Types of Podal Adhesion

There were obvious differences in the distribution of adhesion structures on the tarsi of the anterior, middle and hind legs of adults, and there were also certain differences between males and females. The female fore-leg tarsus has three kinds of adhesion structures, namely, disc-shaped setae, lanceolate setae and serrated setae, while the adhesion structures of the male fore-leg tarsus are obviously different, namely, spatula-shaped setae, lanceolate setae and mushroom-shaped setae. Both males and females have lanceolate bristles on the first tarsus of the fore-leg, but the males have more mushroom-shaped bristles than the females. Both males and females have lanceolate bristles on the second tarsus of the fore-leg, but the females also have serrated bristles, and males have spatula-shaped setae on the third tarsal segment of the fore-leg. Females have disc-shaped setae, while males have spatula-shaped setae.

The differences in adhesion structures of the tarsus of the mid-leg between males and females are also relatively obvious. The first tarsus has only lanceolate setae; the second tarsus of the mid-leg has lanceolate setae and serrated bristles in females, while that in males has lanceolate setae. In addition to the lanceolate setae, there are also spatula-shaped setae; the 3rd tarsus of the mid-leg in both males and females is similar to the 3rd tarsus of the fore-leg in terms of the distribution of adhesion structures, but the types are completely different. Those of the females have disc-shaped setae, while those of the males have spatula-shaped bristles; the hind-leg tarsus of both males and females consists of disc-shaped setae and lanceolate bristles, and the distributions are also the same: the 1st and 2nd tarsi of the hind-legs.

### Transcriptome differences of adhesion organs

#### Screening of differentially expressed genes

**Fig 6:**
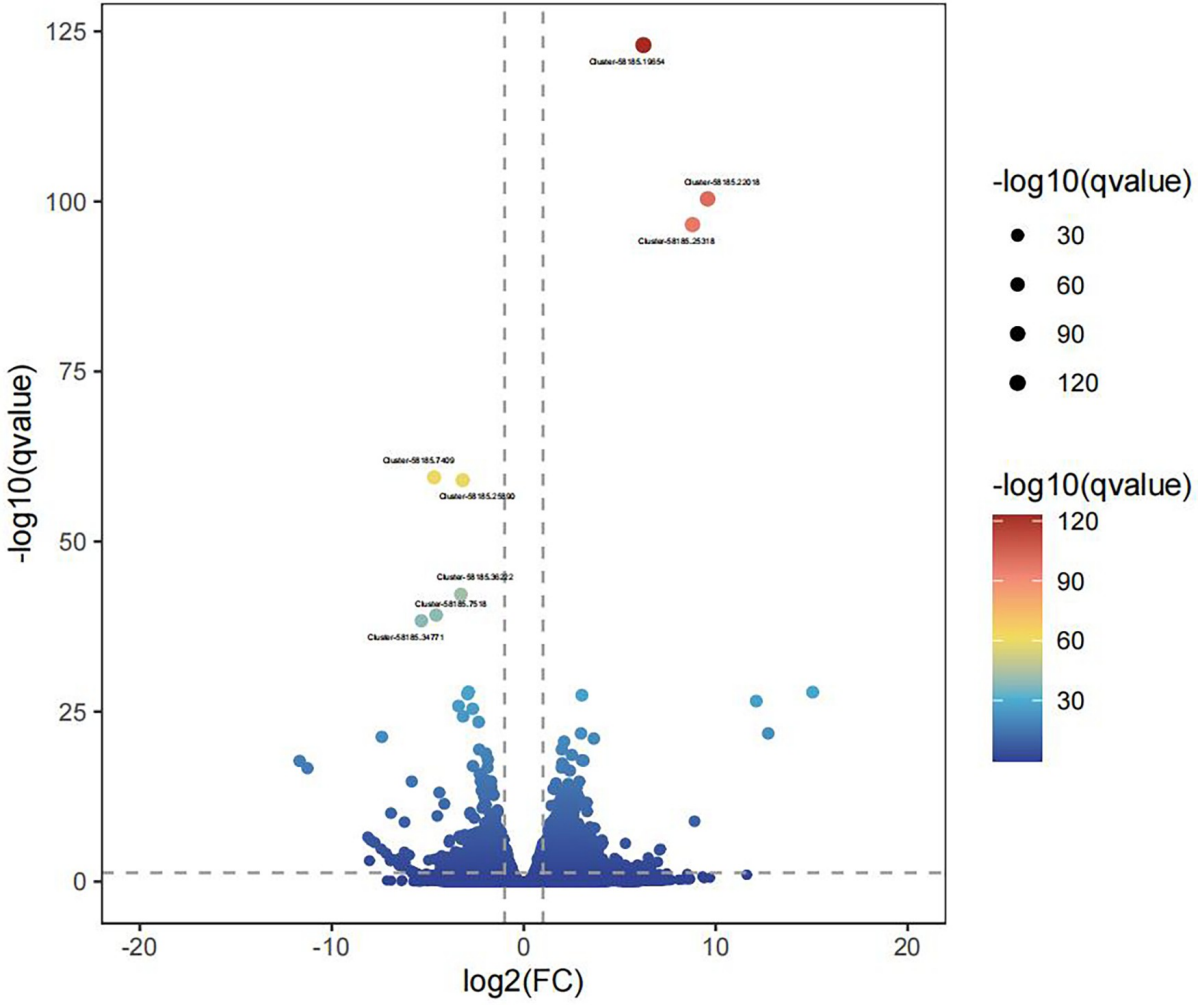
Volcano plot of differentially expressed genes for transcriptome sequencing between the female legs and male legs in adult *G*. *cantor*.

The number of differentially expressed genes (DEGs) between the legs of female and male adults was determined to be 1926 by screening conditions of *q* value < 0.05 and |log2(FoldChange)| > 1, of which 1283 were upregulated and 643 were downregulated (Fig 6). The most significantly upregulated DEG was Cluster-58185.19654, which was annotated as actin β/γ1 and increased in expression by 75.129 times. Other upregulated DEGs were Cluster-58185.22018 and Cluster-58185.25318, annotated as lipase 3, which is closely related to lipid metabolism. The most significantly downregulated DEG (Cluster-58185.7409) had a fold change = 0.039, and the gene was annotated as a structural constituent of the cuticle. Another downregulated gene was a putative glucosylceramidase, which is involved in the glycosphingolipid metabolic process.

Table 5 shows some relevant differentially expressed genes in the female-leg and male-leg groups, including 10 olfaction-related genes and 3 mechanical sensing genes. Among them, females showed upregulation of OBP 1 (fold change = 3.528), OBP 2 (fold change = 2.035), OBP 3 (fold change = 2.003), OR 3 (fold change = 3.477), GR 1 (fold change = 5.404), MSP 1 (fold change = 5.356), MSP 2 (fold change = 9.074) and MSP 3 (fold change = 3.579). On the other hand, males showed upregulation of OBP 4 (fold change = -0.389), CSP 1 (fold change = 0.396), CSP 2 (fold change = 0.302), OR 1 (fold change = 0.247) and OR 2 (fold change = fold change = 0.177).

**Table 5:** Some relevant differentially expressed genes between the female legs and male legs of adult *G*. *cantor*.

Types	Name	Gene ID	Protein ID	Species	qvalue	FoldChange
Odorant-binding	OBP1	Cluster-58185.4107	ADD82416	Batocera horsfieldi Hope	4.28E-02	3.528
protein	OBP2	Cluster-58185.22085	AXO78385	Xylotrechus quadripes Chervolat	2.52E-05	2.035
	OBP3	Cluster-58185.22792	ADD82416	B. horsfieldi	2.62E-03	2.003
	OBP4	Cluster-58185.22517	AUF72988	Anoplophora chinensis Forster	6.35E-11	0.389
Chemosensoy	CSP1	Cluster-58185.20033	AMP19498	Tribolium castaneum Coleoptera	1.71E-04	0.396
protein	CSP2	Cluster-58185.2338	AUF72993	A. chinensis	1.29E-03	0.302
Olfactory receptors	OR1	Cluster-58185.2437	XP_018567483	Anoplophora glabripennis Motschulsky	1.79E-04	0.247
	OR2	Cluster-58185.2246	ANV97828	A. chinensis	1.13E-06	0.177
	OR3	Cluster-58185.3539	AVN97835	A. chinensis	2.29E-04	3.477
Gustatory receptors	GR1	Cluster-58185.943	AVN97869	Aethina tumida Murray	3.52E-02	5.404
Mechanosensitive	MSP1	Cluster-63417.0	PF00924	Nilaparvata lugens Stdl	1.42E-04	5.356
protein	MSP2	Cluster-63417.1	PF01198	N. lugens	5.95E-03	9.074
	MSP3	Cluster-58185.18036	PF05656	N. lugens	2.82E-02	3.579

Note: Relevant data are presented as female versus male; 9.29E-04 = 9.29 × 10^−4^.

#### Cluster analysis of differentially expressed genes

**Fig 7:**
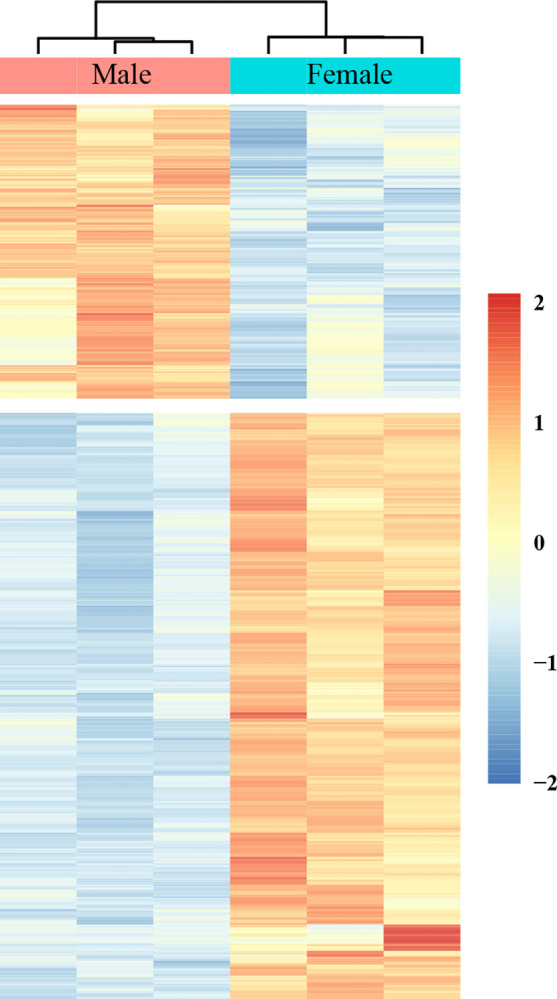
Cluster analysis of differentially expressed genes in the transcriptome of the female legs and male legs of adult *G*. *cantor*.

The method used to determine the clustering pattern of differential gene expression under different experimental conditions was differential gene clustering analysis. In the figure below, red indicates high expression, and blue indicates low expression. The blue and red parts of the test results in this study can be clearly distinguished (Fig 7). Screening of differentially expressed genes showed that there were significant differences in gene expression between the female-leg and male-leg groups. The overall sequencing quality of the samples was good, and they could thus be used in the following analysis.

#### GO enrichment analysis of differentially expressed genes

**Fig 8:**
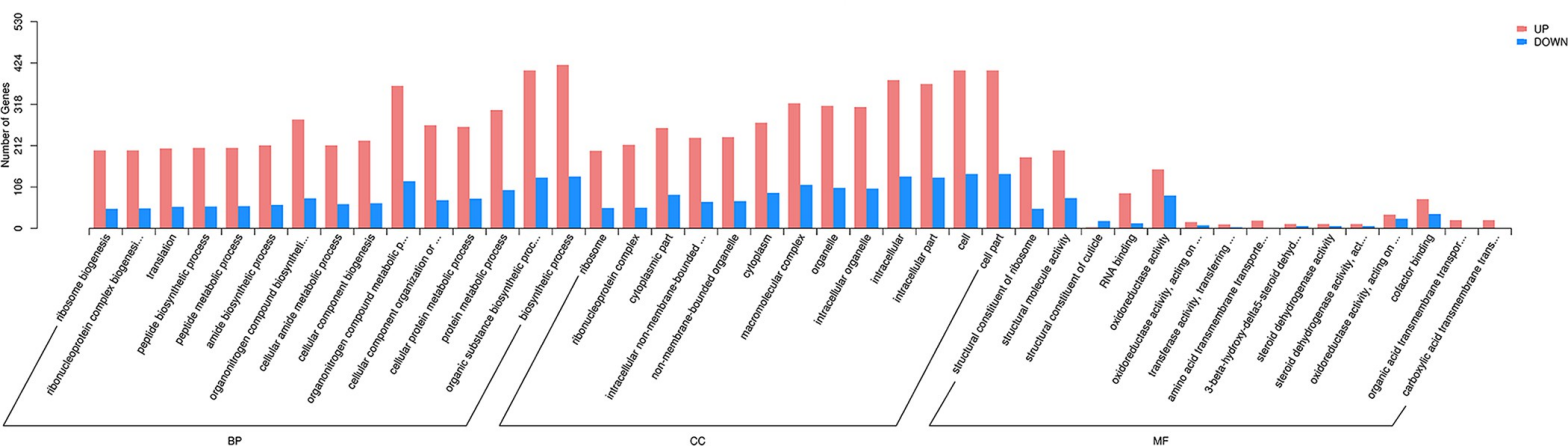
Enriched GO term plot between the female legs and male legs of *G*. *cantor*.

In the female-leg and male-leg groups of adult *G*. *cantor*, GO enrichment analysis was performed on DEGs, and 2638 DEGs were successfully annotated. The DEGs were divided into three categories: biological process, cellular component and molecular function (Fig 8). The biological process category included 1553 items, the cellular component category included 390 items, and the molecular function category included 694 items. The largest number of DEGs was observed for biological processes. In this process, DEGs were found to be enriched in organic substance biosynthetic process (535), biosynthetic process (552), and cellular biosynthetic process (511). Metabolic process came second, and the DEGs were enriched in intracellular (513), intracellular part (500), and cell (544). In the molecular function category, the DEGs were enriched in oxidoreductase activity (235), structural constituent of ribosome (232), and structural molecule activity (278).

Regarding the enrichment significance of GO entries, the top 3 significantly enriched GO terms in the three categories between female legs and male legs of adult *G*. *cantor* were identified. Structural constituent of the ribosome was the most significant (*q* = 8.07E-67), followed by structural molecule activity (*q* = 1.52E-49) (Table 6). On the other hand, the male legs showed the most upregulation in molecular function, structural molecule activity (q = 2.10E-15) and structural constituent of ribosome (*q* = 8.24E-10) (Table 6).

**Table 6:** The top 3 significantly enriched GO terms in three categories between the female legs and male legs of adult *G*. *cantor*.

Types	Term	GO accession	Description	Number of DEGs	qvalue
Up-regulated	biological	GO:0009058	biosynthetic process	419	4.53E-21
	process	GO:1901576	organic substance biosynthetic process	405	9.07E-21
		GO:0044249	cellular biosynthetic process	386	5.83E-19
	cellular	GO:0005623	cell	405	6.04E-10
	component	GO:0044464	cell part	405	6.04E-10
		GO:0005622	intracellular	380	5.43E-10
	molecular	GO:0001598	structural molecule activity	200	1.52E-49
	function	GO:0003735	structural constituent of ribosome	182	8.07E-67
		GO:0003723	RNA binding	90	3.84E-08
Down-regulated	biological	GO:1901564	organonitrogen compound metabolic process	121	9.41E-04
	process	GO:1901566	organonitrogen compound biosynthetic process	77	1.63E-03
		GO:0044085	cellular component biogenesis	64	3.16E-04
	cellular	GO:0005737	cytoplasm	91	9.18E-04
	component	GO:0044444	cytoplasmic part	86	6.37E-06
		GO:0043228	non-membrane-bounded organelle	70	6.03E-03
	molecular	GO:0016491	oxidoreductase activity	84	6.59E-03
	function	GO:0005198	structural molecule activity	78	2.10E-15
		GO:0003735	structural constituent of ribosome	50	8.24E-10

Note: Relevant data are presented as female versus male; 4.53E-21 = 4.53 × 10^−21^.

#### KEGG enrichment analysis of differentially expressed genes

**Fig 9:**
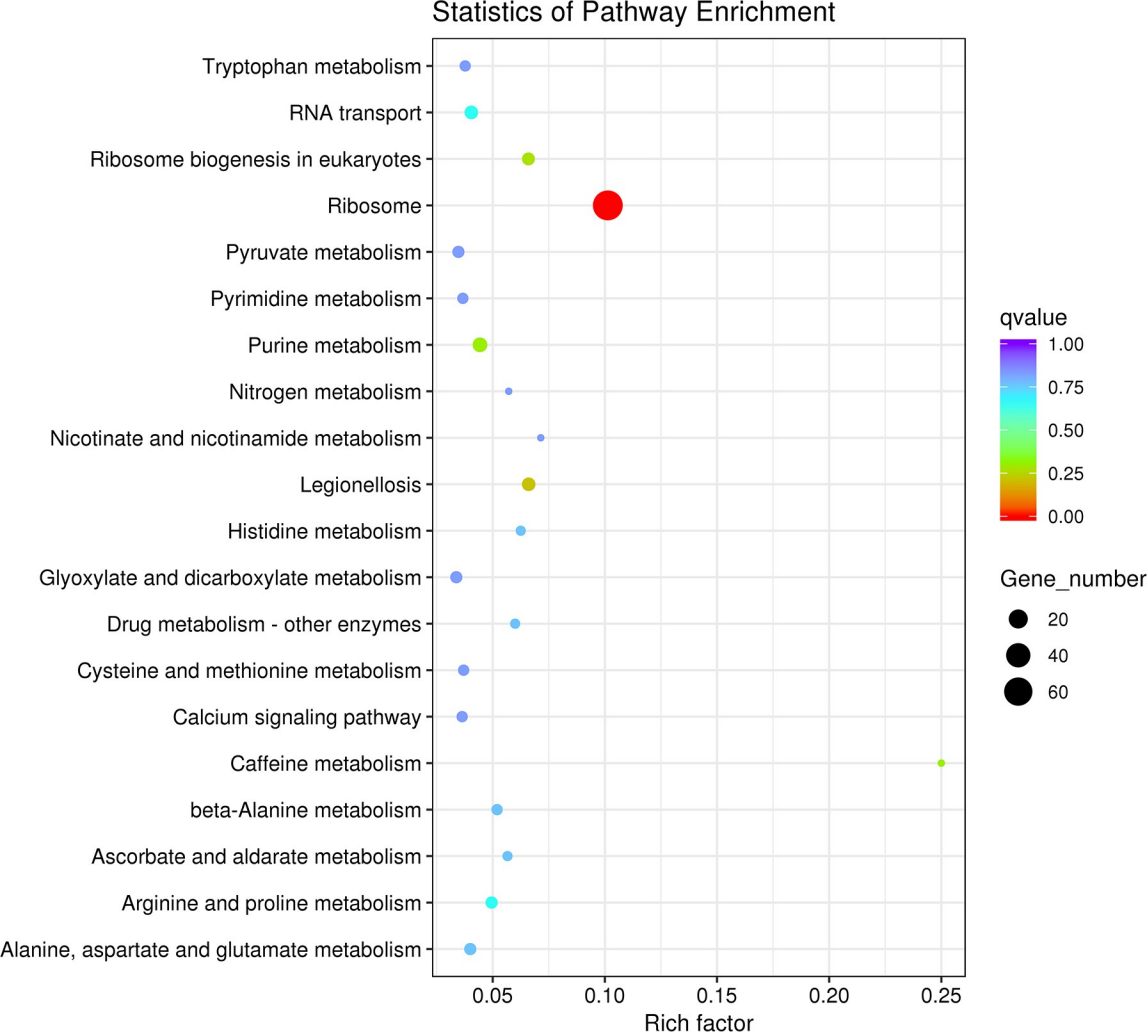
KEGG enrichment analysis of differentially expressed genes between the female legs and male legs of *G*. *cantor*.

The 20 pathway entries with the most significant enrichment were selected and displayed in the map. The results showed that the differentially expressed genes in the legs of the male and female beetles were obvious. The pathway q values are shown, with Ribosome exhibiting the most significant differences (Fig 9). Moreover, Purine metabolism, Arginine metabolism, Proline metabolism and RNA transport also showed significant differences (Fig 9).

## Discussion

The number of beats and posture of the tarsal segments of *G*. *cantor* varied during each behaviour [24], and electron microscopic scanning also revealed some variability in the functional structures. The tarsal segments of the legs of *G*. *cantor* exhibit two primary types of sensilla and adhesion structures in their functional organization. There is no significant variation in the distribution of sensilla between males and females or between the fore-legs, mid-legs and hind-legs. There have been many reports on the sensilla of insects, and their functions and external morphological characteristics have been systematically recorded [27]. The distribution of mechanical sensilla (SCh and Bb) on the joints and abdomen is extensive (Tables 2–4), potentially due to those parts increased involvement in mechanical sensing. This observation aligns with previous studies demonstrating a close correlation between sensor density and sensing function [28]. On the other hand, a role in olfaction and gustation is suggested for ST [27]. Therefore, ST I are distributed on the dorsal surface of the tarsus and play a role in the recognition of external phytochemical odours.

Another functional structure of *G*. *cantor* is the adhesion structure. Many types of adhesion structures have a common basic function of physical shock absorption [29–31]. However, there are obvious differences in the distributions and types on the fore-legs, mid-legs, and hind-legs of each sex of *G*. *cantor*. Different roles are played in various behaviours. The female-unique serrated bristle has many sawtooth-shaped tapered sensilla at the tip, which greatly increase its grip. During the spawning process, a certain degree of thrust is provided [32–34]. In addition, another unique adhesion structure of the female is disc-shaped bristle, and its function is mainly adhesion, and the contact area of the section and the medium is increased. This structure is also covered with a large number of cone-shaped spines, which further increase its adhesion to provide greater attachment during the expression of pre-spawning behaviour [32,35,36]. Some studies have shown through electrophysiological experiments that there are contact chemical sensilla on the legs of insects, which can not only perceive salt, water, sugar, and alkaloids [37,38] but also perceive the host extract [39] and act as a spawning stimulator [40]. Through ultramicrostructural observation, it was found that the sawtooth-shaped rigid discs are tapered to a point, which is very similar to the shape of the spore sensillum (SG) on the ovipositor [28]. Therefore, the role of chemical sensilla is also similar. However, few relevant research reports have been published, so this problem needs to be further addressed through experiments. In addition, it is also reasonable to speculate that these olfactory perception functions and mechanical functions are related to the sensillum and adhesion structures of the legs and to the chemosensory genes of the legs.

Therefore, we performed more in-depth analysis to examine the chemosensory genes in the legs. We found significant differences between the female legs and male legs of adult *G*. *cantor* by RNA-seq technology. Through comparative analysis, we screened a subset of olfaction- and mechanics-related genes with significant variability [41]. The male legs showed upregulation of 1 OBP, 2 ORs and 2 CSPs. A large number of studies have shown that these three types of proteins are directly related to the recognition of sex pheromones [42–44].

The mating behaviour of *G*. *cantor* has been studied in detail, and it has been confirmed that the male reacts to the female with his antennae or tarsi [45–49], and the male dashes to the female and mounts her back (Fig 1A). According to the results of this experiment, tarsi play an important role in the process of sex pheromone recognition [50,51]. On the other hand, the female legs showed upregulation of 3 OBPs, 1 OR, 1 GR and 3 MSPs. As previously mentioned, OBPs, ORs and GRs are also involved in the olfactory recognition process but are more involved in the recognition of oviposition pheromones. Similar functions have been reported in other insects, such as Drosophila suzukii and Plagiodera versicolora [41,47]. Interestingly, the study also showed sex differences in MSPs. Although a large number of studies have shown that insect bristles are model mechanosensory organs [52–54], research on MSPs is scarce. Research shows that mechanosensitive channels in insect bristles are specialized for detecting mechanical forces generated by touch, the stretching of body parts, gravity or sounds [55,56]. It has been reported that *G*. *cantor* mainly relies on its tarsal spiny sensilla to sense the grasping force of the tarsal ganglion and identify the roughness of the host surface (Fig 1B). This result reveals why MSP differences occur.

In summary, this study was designed to characterize external morphology and gene expression using SEM and transcriptomics, which provided valuable insights into the possible functional differences between the legs of different sexes of *G*. *cantor*. We evaluated sexual differences in the type and distribution of various sensilla and adhesion structures in three pairs of legs. Moreover, we also analysed the differences in olfactory and mechanical functions between male and female legs at the genetic level. These results will help us better understand the host selection and courtship behaviours of *G*. *cantor* and help correlate these behaviours with electrophysiological mechanisms in future studies. The observed differences in ultrastructure and gene expression will greatly facilitate the design of more effective semiochemical control methods for insect pests.

## Conclusions

Insect legs show unique structures and physiological functions during development, which determine the different reproduction modes of insects. *G*. *cantor* is one of the most important pests of forests, trees and fruits, and we investigated the leg organs that affect the reproductive behaviour of this pest for the purpose to enrich the prevention and control methods of this pest. The present study aimed to characterize possible sexual and functional differences in *G*. *cantor* legs using SEM and transcriptome techniques, thereby providing valuable insights into external morphology and gene expression.

We performed a systematic study of the ultrastructure and gene expression of the legs of *G*. *cantor* and assessed sex differences in different sensilla and adhesion structures in three pairs of legs. The adhesive structures of the fore and middle legs of the female longhorn beetle are similar, which help in grasping and adhering during the egg-laying process, while the hind legs mainly serve a supporting role. In addition, we also performed genetic analyses of the olfactory and mechanical functions of male and female legs to explore their differences. We discovered that the male legs showed upregulation of 1 OBP, 2 ORs and 2 CSPs. These up-regulated genes influence clutching behaviour in males. Meanwhile, the female legs showed upregulation of 3 OBPs, 1 OR, 1 GR and 3 MSPs. These genes are clearly involved in the female’s egg-laying behavior. These results will help us better understand host selection and courtship behaviours and help us interrelate these behaviours with electrophysiological mechanisms. Research on the ultrastructure and gene expression differences of the legs of pests can greatly aid in the design of chemical pest control methods.

## Supporting information

S1 File Supplement tables. (XLSX)
